# Metabolic Fingerprinting of Feces from Calves, Subjected to Gram-Negative Bacterial Endotoxin

**DOI:** 10.3390/metabo11020108

**Published:** 2021-02-13

**Authors:** Saeid Kamel Oroumieh, Abbas Ali Naserian, Lieven Van Meulebroek, Ellen De Paepe, Reza Valizadeh, Lynn Vanhaecke

**Affiliations:** 1 Laboratory of Chemical Analysis, Department of Veterinary Public Health and Food Safety, Faculty of Veterinary Medicine, Ghent University, Salisburylaan 133, 9820 Merelbeke, Belgium; Saeid.Kameloroumieh@Ugent.be (S.K.O.); Lieven.Vanmeulenbroek@Ugent.be (L.V.M.); Ellen.Depaepe@Ugent.be (E.D.P.); 2 Department of Animal Science, Faculty of Agriculture, Ferdowsi University of Mashhad, Mashhad P.O. Box 91775-1163, Iran; Naserian@um.ac.ir (A.A.N.); Valizadeh@um.ac.ir (R.V.)

**Keywords:** ultra-high performance liquid-chromatography high-resolution mass spectrometry, fecal metabolomics, biomarker, Gram-negative bacterial endotoxin, inflammation

## Abstract

Gram-negative bacteria have a well-known impact on the disease state of neonatal calves and their mortality. This study was the first to implement untargeted metabolomics on calves’ fecal samples to unravel the effect of Gram-negative bacterial endotoxin lipopolysaccharide (LPS). In this context, calves were challenged with LPS and administered with fish oil, nanocurcumin, or dexamethasone to evaluate treatment effects. Ultra-high-performance liquid-chromatography high-resolution mass spectrometry (UHPLC-HRMS) was employed to map fecal metabolic fingerprints from the various groups before and after LPS challenge. Based on the generated fingerprints, including 9650 unique feature ions, significant separation according to LPS group was achieved through orthogonal partial least squares discriminant analysis (Q^2^ of 0.57 and *p*-value of 0.022), which allowed the selection of 37 metabolites as bacterial endotoxin markers. Tentative identification of these markers suggested that the majority belonged to the subclass of the carboxylic acid derivatives—amino acids, peptides, and analogs—and fatty amides, with these subclasses playing a role in the metabolism of steroids, histidine, glutamate, and folate. Biological interpretations supported the revealed markers’ potential to aid in disease diagnosis, whereas beneficial effects were observed following dexamethasone, fish oil, and nanocurcumin treatment.

## 1. Introduction

Calf mortality causes significant economic loss and has therefore been distinguished as an index of dairy farm health status; however, reducing calf mortality remains a vital challenge in dairy cow herds [1]. In this context, Gram-negative bacteria have a well-known impact on the cause of neonatal diarrhea, bovine respiratory disease (BRD), and septicemia, which are the most pertinent calf diseases causing mortality [2,3,4]. These Gram-negative bacteria release the endotoxin lipopolysaccharide (LPS) through bacterial proliferation or cell death and lysis. LPS is responsible for major clinical issues in cattle, such as endotoxemia and Gram-negative sepsis [5]. Due to the speedy pathogenesis of Gram-negative bacterial infections in calves, an early overcoming is critical since late treatment is typically not adequate to alter the disease process [5]. The designated treatment of these acute inflammatory diseases includes (non)-steroidal anti-inflammatory drugs, with the glucocorticoid dexamethasone being widely used due to its significant short-term effects on the acute phase response (APR), despite of its immunosuppressive effect [5]. Failure to treat the disease can be contributed to two main factors; (1) rapid progression fast of the disease and associated inflammatory response and (2) late recognition by farmers. Therefore, rapid disease identification and early treatment in calves is crucial. Unfortunately, the early diagnosis of sepsis in calves is still a big challenge as the disease is characterized by multiple, non-specific clinical signs. The only certain diagnosis of septicemia is based on blood culture tests, for which the results are only available in a late stage (after 42–72 h). Additionally, false-negative culture findings may occur as many factors could interfere with bacterial isolation from a blood culture [6,7]. To conclude, most of the current laboratory investigations are not sufficiently specific to sepsis, thereby necessitating a combination of different technological and methodological approaches to integrate the clinical symptoms of the septic animal [6,7]. 

In this regard, metabolomics has surfaced in recent years as a promising methodology, reflecting the interactions of a calf’s genes with epigenetic factors such as environment and feeding regime, thereby accurately representing its phenotype [7,8]. For a particular physiological state and under specific environmental conditions, metabolomics covers a wide range of metabolites manifested in the biological system under investigation [9,10]. Unlike the genotype, the metabolic phenotype is not stable over time, with metabolites and their abundances changing according to metabolic state. As such, metabolomics may support the identification of biomarkers that can be used for disease diagnosis or even risk prediction, as well as to unravel (patho)physiological pathways [11,12,13]. In this regard, LC-MS technology enables high-level separation and characterization of metabolites, thereby providing accurate (semi-)quantification and identification [14]. On the subject of calves’ disease, metabolomics has already been performed, although solely focusing on plasma and/or serum and implementing targeted metabolomics [7,15,16,17,18]. These particular studies assessed diarrhea, BRD, and sepsis in calves, whereby creatine, choline, acetate, lysine, 2-methylglutarate, allantoin, and phenylalanine were ascertained to be significantly affected by health state and therefore introduced as potential biomarkers. 

In the current study, an LPS challenge model introduced by Plessers et al., 2015 [19] was applied to induce an APR in calves. The latter can be considered as a valuable challenge experiment towards the exploration of metabolic alterations, as caused by Gram-negative bacterial infections. To the best of our knowledge, this research is the first to perform untargeted metabolomics in the quest to define fecal metabolites that are significantly affected by Gram-negative bacterial endotoxins (LPS challenge) in calves. Additionally, this study performed pioneering work on the effect of dexamethasone as compared to natural inflammatory compounds (fish oil and curcumin) on disease progression following LPS-challenge as well as on the previously described fecal metabolite markers. Hereby, fish oil and curcumin were selected since the inclusion of these natural anti-inflammatory supplements in animals’ diets have been suggested to improve health status [20,21,22]. 

Metabolic fingerprinting of fecal samples was conducted by untargeted ultra-high-performance liquid chromatography coupled to hybrid high-resolution orbitrap mass spectrometry (UHPLC-HR-Q-Orbitrap-MS), based on a validated protocol for human fecal samples [11,23]. The fecal metabolites of Gram-negative bacterial infections under APR were identified using chemometric data analysis and may serve as new biomarkers for early-phase diagnosis of calves’ diseases (Gram-negative bacterial infections) and treatment efficacy.

## 2. Results

### 2.1. Acute Phase Response

The clinical results of the APR experiment have been reported previously [24]. In brief, repeated measures ANOVA revealed that rectal body temperature (RT), heart rate (HR), respiratory rate (RR), and cytokines were significantly altered (*p*-value < 0.05) within each group, before and after LPS challenge. No significant (time) effects were observed within the CON group. Additionally, both RT and HR were significantly different at 2–18 h post LPS challenge (p.c.) (*p*-value < 0.05) between CON and other groups, while at 0.5–2 h p.c. a significant difference could also be observed in RR (*p*-value < 0.05). Concentrations of TNF-α and IL-6 in LPS challenged groups were significantly increased between 1-6 h p.c. (*p*-value < 0.05). The areas under the curve for RT, HR, RR, TNF-α, and IL-6 in the different groups, are presented in Supplementary Table S2. These results confirmed APR in calves through Gram-negative bacterial endotoxin exposure. 

### 2.2. Metabolic Fingerprinting by UHPLC-HRMS

**Figure 1:**
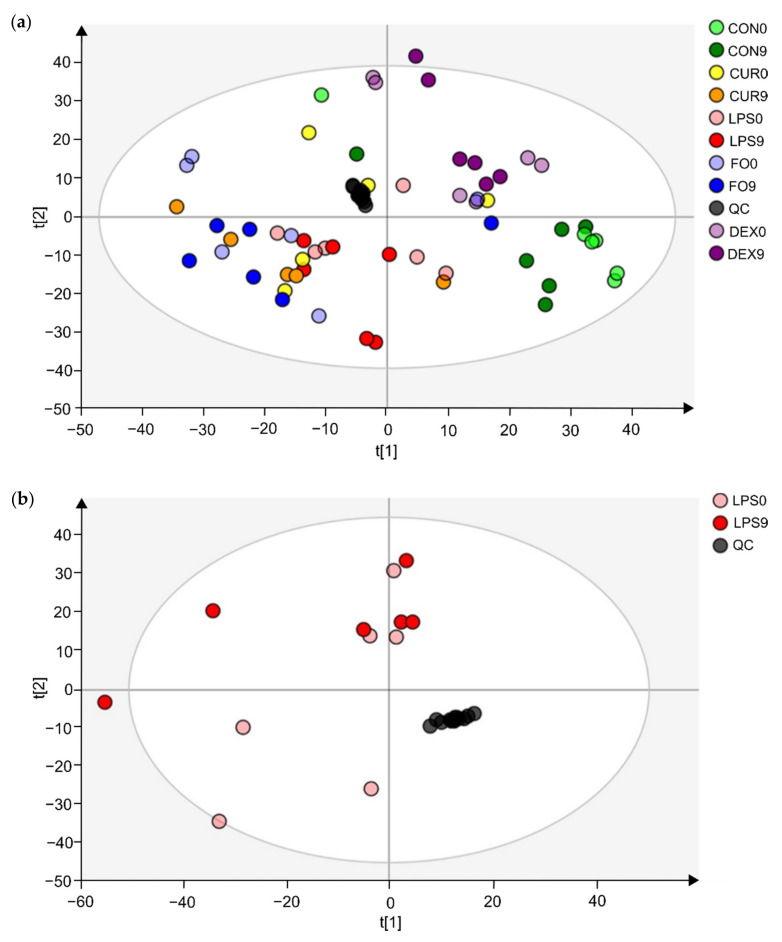

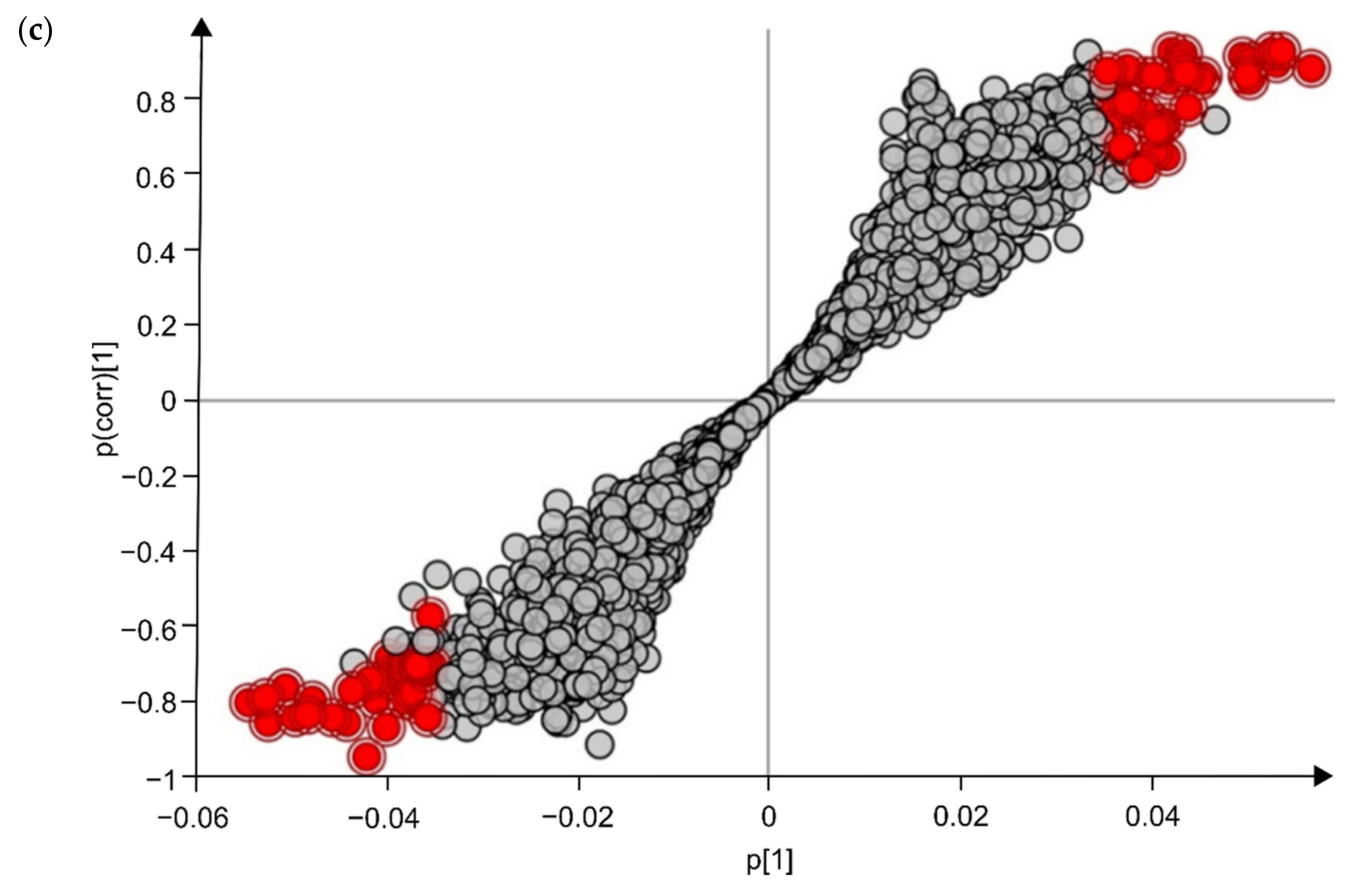
Multivariate statistical analysis plots of LPS challenged calves, for which fecal metabolomics was applied, using the combination of positive and negative ionization modes. (**a**) The PCA-X model’s score plot for the control samples (CON) and LPS challenged calves’ feces samples (LPS, FO, CUR, DEX). (**b**) The PCA-X model’s score plot of LPS day 0 and LPS day 9. The light and dark colors represent days 0 and 9, respectively. (**c**) S-plot for the OPLS-DA model, comparing day 0 and day 9 for the LPS group samples, wherein each dot represents a metabolite. The metabolites (*n* = 78) shown in red were retained for further analysis.

Untargeted metabolomics analysis revealed 9650 unique feature ions for the combination of positive and negative ionization. PCA-X modeling displayed good clustering of QC-samples (Figure 1a). For the LPS group, it was not possible to construct a valid OPLS-DA model (day 0 vs. day 9) based on the entire set of metabolites, possibly indicating the presence of noise data. Therefore, data were pre-filtered based on a VIP ≥ 1, which rendered a subset of 3132 feature ions potentially involved in the APR. Based on this subset, natural clustering according to APR state (LPS group, day 0 vs. day 9) was not clear in the unsupervised model (Figure 1b), indicating that other sources of variation were more predominant. However, supervised OPLS-DA modeling suggested significant metabolic differences according to APR classification, which was confirmed by valid model parameters; R^2^Y of 0.86, Q^2^ of 0.57, valid permutation testing (*n* = 100), and CV-ANOVA *p*-value of 0.022. In addition, an OPLS-DA model was constructed for the CON group (day 0 vs. day 9), considering the subset of 3132 ions, of which no valid model could be established (R^2^Y of 0.98, Q^2^ of 0.56, valid permutation testing (*n* = 100), and CV-ANOVA *p*-value of 0.166). This finding indicates that the selected fraction of 3132 features ions were truly involved in the APR and were most likely not related to a (sampling) time-effect (day 0 vs. day 9). As such, the OPLS-DA model as constructed within the LPS group was further used to select the most promising metabolite biomarkers, based on the VIP-score (>1), Jack-knifed confidence interval (not across zero), and S-plot data (correlation |pcorr| > 0.5, covariance |p| > 0.035) (Figure 1c). This allowed the selection of 78 feature ions, for which an additional t-test analysis was performed, again for both the LPS group (day 0 vs. day 9) and CON group (day 0 vs. day 9) to eliminate time-effect and assure all metabolites were related to APR. As such, 37 feature ions were retained as potential Gram-negative bacterial endotoxin markers. 

Subsequently, the effect of dexamethasone, fish oil, and curcumin treatment on the potential endotoxin markers was evaluated using a *t*-test on the abundance shifts, i.e., LPS |day 0–day 9| was compared to the other three groups |day 0–day 9|. This revealed a significant impact on, respectively 24, 10, and 11 potential biomarkers (*p*-value < 0.05). It may be concluded that the impact of dexamethasone treatment was most prominent, as could be expected, but that other treatment also attained some beneficial effects. 

### 2.3. Identification of Potential UHPLC-HRMS Metabolite Markers

**Figure 2:**
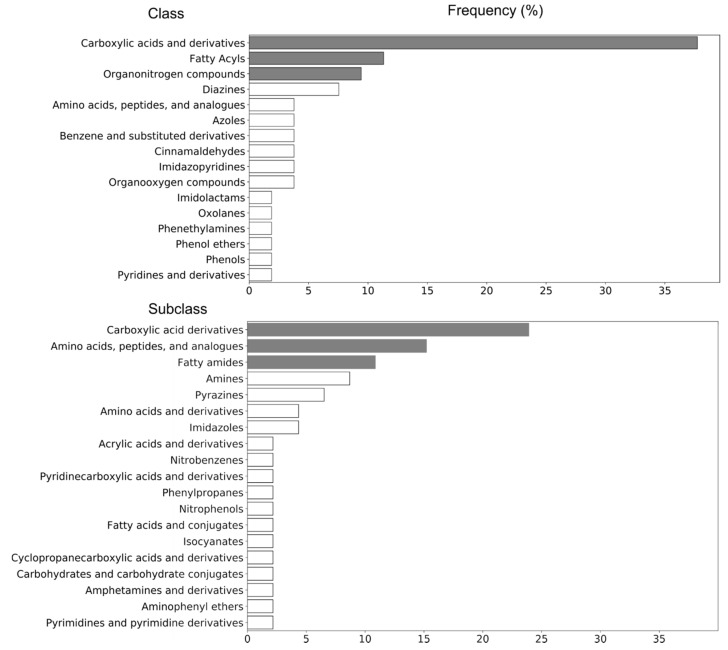
Structural categorization of potential fecal markers (*n* = 37) for LPS challenge, according to the ClassyFire classification system.

The defined markers (*n* = 37) were tentatively identified by taking into account retention time, accurate mass (MS^1^) and precursor-specific fragmentation data (MS^2^) [14]. The Gram-negative bacterial endotoxin biomarkers and associated chemical data (including proposed chemical formula and structures, as obtained by SIRIUS software) are presented in Table 1 and Table 2. In addition, chemical structures and identities (IUPAC-name and PubChem ID), as well as chemical taxonomy, are presented in Supplementary Table S1. The class and subclass frequency of the markers, derived from Table 1 and Supplementary Table S1, are shown in Figure 2. Carboxylic acids and derivatives, fatty acyls, and organonitrogen compounds were the top-ranked classes, while carboxylic acid derivatives—amino acids, peptides, and analogues—and fatty amides were the most frequently represented subclasses. 

For eight metabolites (IDs: 21, 22, 27, 29, 31, 32, 35, and 36) no relevant fragmentation data could be obtained due to low intensities or interfering isobaric compounds; therefore, no structural elucidation could be suggested for these metabolites (Supplementary Table S1). In addition, for seven markers, no candidate structures were retrieved by SIRIUS software, although fragmentation data were available (IDs, 3, 6, 8, 13, 15, 16, 37; Supplementary Table S1). 

### 2.4. Identification of Potential Pathway Alterations

**Figure 3:**
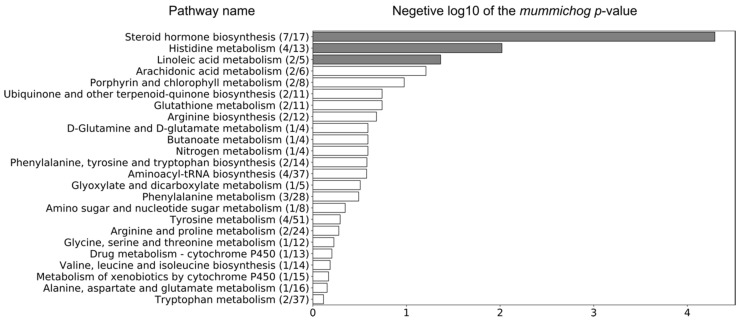
Three metabolic pathways were significantly altered in the LPS group between day 0 and 9, based on the 3132 ions that were obtained by pre-filtering the data (VIP > 1) using the associated OPLS-DA model (grey bars represent significantly altered metabolic pathways, *p*-value < 0.05). The ratio in brackets represents the number of significant matches to the total number of reported metabolites in the pathway.

**Figure 4:**
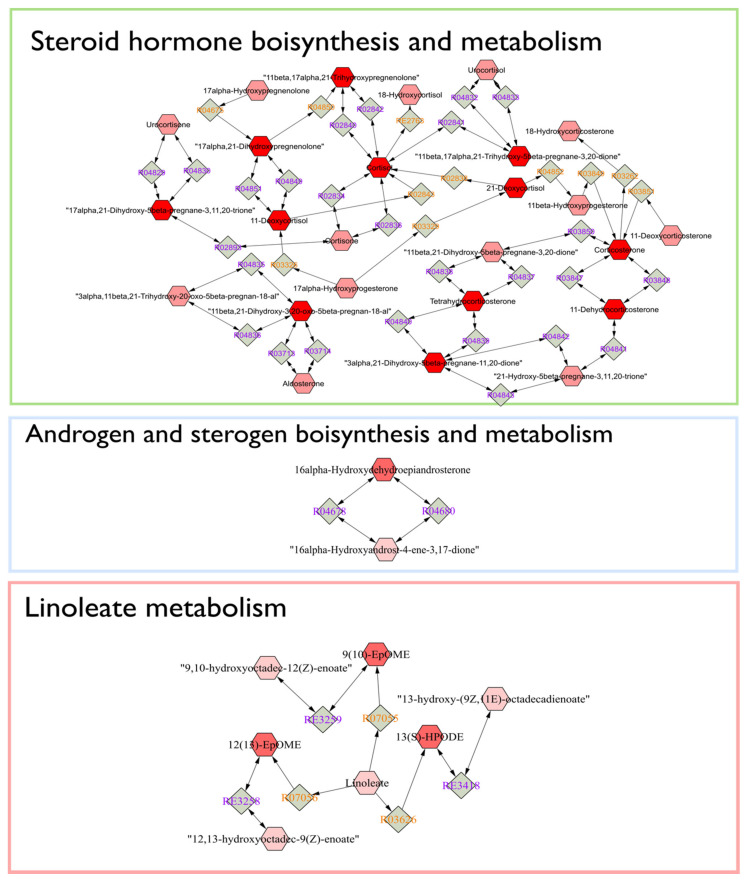

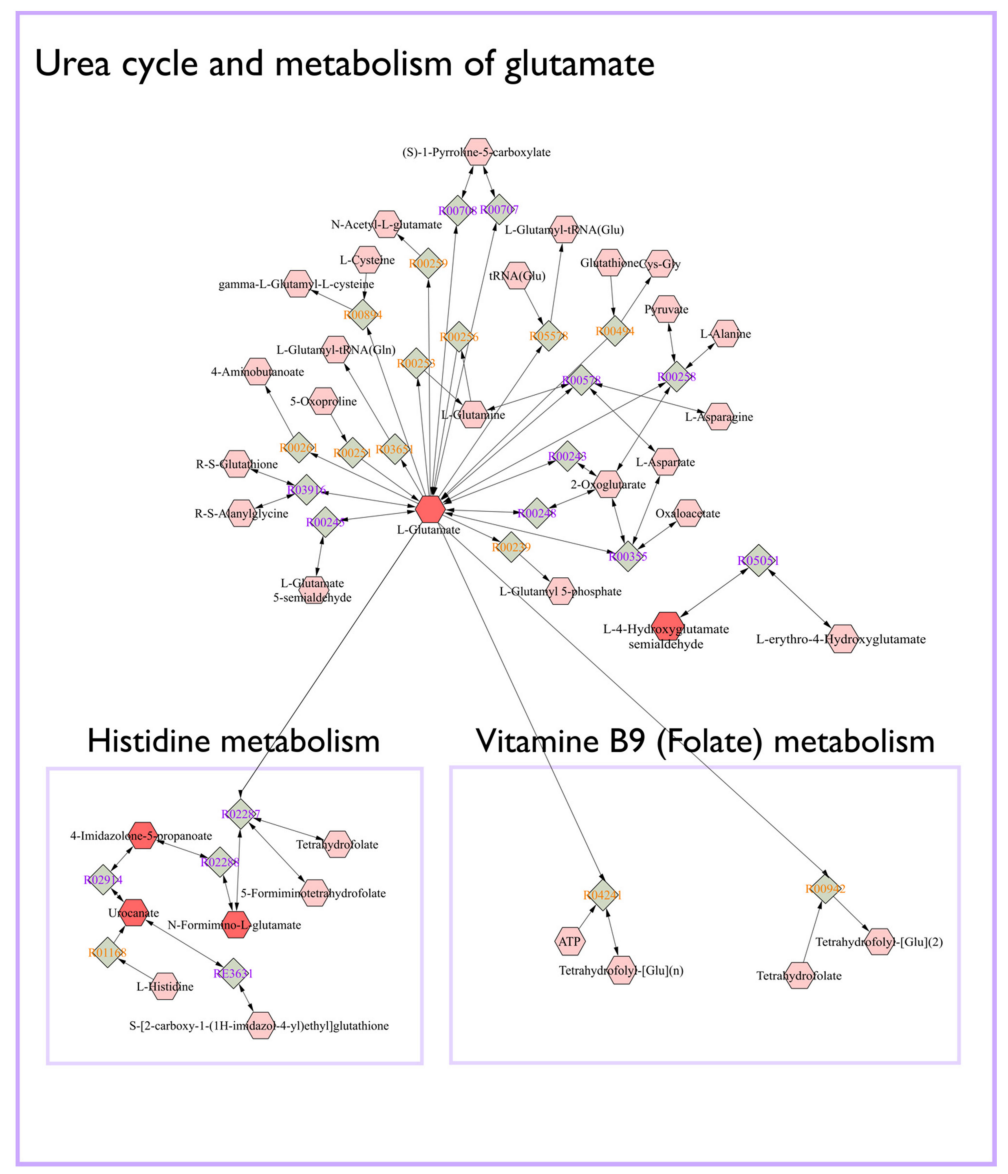
Connected networks of metabolites in the significant pathways based on the KEGG database. The nodes in red indicated differential metabolites that could be measured in this study.

The Mummichog algorithm identified three metabolic pathways (Figure 3) that were significantly altered in LPS group between day 0 and 9, based on the 3132 ions (Figure 1b). The steroid hormone biosynthesis pathway was most significantly altered, followed by the histidine and the linoleic acid metabolism (Figure 3). Subsequently, a connected network of metabolites was built based on the KEGG identifiers of the metabolites belonging to the three selected pathways, using Metscape [25] as a plug-in for Cytoscape [26] (Figure 4). This revealed that the metabolism of α-amino acids such as histidine and glutamate, the urea cycle, folate, steroid hormones, and linoleate metabolism were connected to the Gram-negative bacterial endotoxins’ markers (Figure 4).

## 3. Discussion

**Figure 5:**
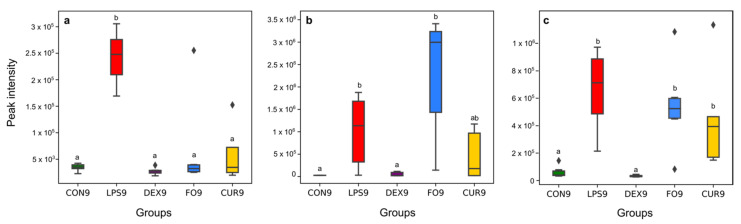
Effect of LPS, DEX, FO, and CUR on selected biomarkers, highly representative for the carboxylic acid derivatives subclass ((**a**), ID23), fatty amides subclass ((**b**), ID17), and steroid hormone biosynthesis and metabolism ((**c**), ID31). The different letters indicate a significant difference between groups (*p*-value < 0.05).

UHPLC-HRMS data interpretation revealed that carboxylic acid derivatives—amino acids, peptides, and analogs—and fatty amides subclasses might have the most prominent biological role in calves’ diseases, caused by Gram-negative bacterial endotoxins. The results indicated that carboxylic acids and their derivatives have the highest frequency among the chemical compound classes and subclasses of Gram-negative bacterial endotoxins biomarkers (Figure 2). As a class of organic compounds, carboxylic acids contain a carboxyl group attached to the hydrocarbon radical. They are widely distributed in nature and are degradation intermediates from amino acids, fats, and carbohydrates [27]. In the current study, among the biomarkers of carboxylic acid’s subclass, 83% of metabolites increased following LPS challenge, whereby ID23 (Supplementary Table S1, top-ranked structure *N*-[(1R,4R)-4-(propionylamino)-1-methylpentyl]propionamide) could be considered as the most valuable Gram-negative bacterial endotoxin biomarker with considerable differences in abundance within LPS group (LPS0 vs. LPS9: *p*-value of 7.581 × 10^−6^ and 8.29 fold-change, Table 1), and between the LPS and other groups (Figure 5a).

Since carboxylic acids are intermediate metabolites of major organic cellular components, their excess amount in different fluids of the human body have been related to various diseases [28]. Indeed, the accumulation of carboxylic acids indicates organic acidurias, which have been linked to different disorders of protein metabolism. The toxic accumulation of such metabolites, which are generally not present under physiological conditions in the organism, results in an intoxication-like clinical condition. Our results are in line with Nakamura et al., 1993, who reported that the short-chain carboxylic acid concentration was significantly surged in fecal material of patients suffering from chronic pancreatitis [29]. These short-chain carboxylic acids are known to stimulate the inflammatory response by cytokine release [30]. At the cellular level, they inhibit proliferation of epithelial and endothelial cells and hinder leukocyte apoptosis and its function, while they stimulate the release of leukocyte cytokine. At the molecular level, these acids can stimulate neutrophil gene transcription, translation, and protein expression [30]. An in vitro LPS challenge experiment revealed that carboxylic acids, more specifically 5,6-dihydroxyindole-2-carboxylic acid, are potent and specific enhancers of nitric oxide production by LPS-activated murine macrophages [31]. Thus, the likelihood is high that these acids can promote and prolong inflammation. A survey on children with different intestinal microbial states showed that the number of carboxylic acids excreted in feces strongly depended on the degree of the intestine’s microecological imbalance [32]. Additionally, it was demonstrated that the amounts of carboxylic acids in feces were correlated with the degree of the microecological imbalance, induced by LPS challenge [33,34]. With this rationale, the (semi-)quantification of carboxylic acids in calves’ feces samples may support early diagnosis of various diseases and associated metabolic perturbations, i.e., neurotransmitter metabolism, cellular energy, gastrointestinal function, mitochondrial metabolism, and amino acid/organic acid balance [27]. In the current study, dexamethasone, fish oil, and curcumin treatment reduced the impact of LPS challenge for various endotoxin biomarkers. For example, for ID23 (the most promising carboxyl acid marker), the shift that was typically caused by LPS challenge was reduced by 94% in the case of dexamethasone, 75% in the case of fish oil, and 34% in the case of curcumin treatment (Figure 5a). This result may indicate that the various treatments exerted anti-inflammatory effects on the carboxylic acids and derivatives subclass of Gram-negative bacterial endotoxin biomarkers.

Fatty amides, the condensation product of a fatty acid and an amine, represented a second highly reported subclass among the Gram-negative bacterial endotoxin biomarkers (Figure 2). They belong to the lipid-like molecules superclass [35]. In the current study, all metabolite markers assigned to the fatty amides subclass increased upon LPS challenge, with ID17 showing the most pronounced shift (*p*-value of 0.013 and fold-change of 15.15) (Figure 5b). The most well-known physiological effect of fatty amides is their sleep-inducing properties [24,36], which might correlate with depression behavior in response to LPS challenge. Additionally, recent literature has shown that fatty amides, through interactions with unique receptors (extracellular and intracellular), can induce various effects such as appetite suppression, modulation of lipid and glucose metabolism, vasodilation, cardiac function and inflammation [19,24,37]. Finally, it has been reported by Maccarrone et al., 2001 that LPS increases the level of endogenous fatty amides such as the cannabinoid anandamide in rat and human lymphocytes. More specifically, LPS was concluded to hinder the activity of the fatty acid amide hydrolase (a mammalian integral membrane enzyme that degrades fatty acid amides) by downregulating on gene expression at the transcriptional level [38]. There was no significant effect of fish oil and curcumin on the level of ID17, while this metabolite was significantly decreased upon dexamethasone treatment (96%) (Figure 5b). This result confirms our earlier findings [24] that there was no effect of fish oil and curcumin on appetite, sickness behavior, and inflammation response following LPS challenge in calves. In contrast, dexamethasone improved sickness behavior and inflammation response in LPS challenged calves [24].

Based on the Mummichog analysis, steroid hormone biosynthesis and metabolism (Figure 3) was revealed as one of the most important biochemical pathways involved in APR upon LPS-challenge. In this regard, although no useful fragmentation data could be obtained for ID31 (Table 1), this metabolite was probably involved as a cortisol-like structure based on MS^1^ and retention time. Hereby, the comparison was made with the authentic reference standard of cortisol included in the standard mixture that was run at the beginning of the sequence. The concentration of ID31 was significantly increased by the LPS challenge (Figure 5c). Sierra et al., 2008 reported a dramatic down-regulation of steroid receptors (especially glucocorticoid, mineralocorticoid, and estrogen receptors) in microglia shortly after inflammation, induced by systemic injection of LPS. More specifically, LPS induces amongst others the secretion of glucocorticoids, thereby inhibiting the expression of the glucocorticoid receptor [39]. Sierra et al., 2008 hypothesized that the downregulation of steroid hormone receptors, induced by LPS, would let microglia get into a full inflammatory state, which inhibit the cell to retain the authorization of endogenous anti-inflammatory modulators (e.g., steroid hormones) [39]. It is confirmed that LPS significantly increased the level of IL-6 and TNFα in calves [5,19,24]. Im et al., 2012 showed that these cytokines significantly enhanced steroid sulfatase expression (mediating the conversion of estrone sulfate to estrone), which can stimulate growth in endocrine-dependent tumors in human prostate cancer cells [40]. In our study, an anti-inflammatory effect of dexamethasone on ID31 was observed (98%), which might be related to the three independent anti-inflammatory mechanisms for dexamethasone: (1) the induction and activation of annexin I, (2) the induction of MAPK phosphatase 1, and (3) the repression of transcription of NF-κB [41]. In this study, the significant increase of cytokines such as IL-6 and TNFα after LPS challenge, and the Mummichog analysis results may appoint steroid hormones and their derivatives as valuable markers for Gram-negative bacterial endotoxins in calves’ feces.

The other biochemical pathways significantly affected by the LPS challenge enclosed histidine and glutamate metabolism and the urea cycle (Figure 4). Interestingly, histidine and glutamate both are α-amino acids, which were also the most frequently represented parent level in the subclass of amino acids, peptides, and analogues (Supplementary Table S1). Our results align with previous studies that reported glutamate/or glutamine metabolism and the urea cycle to be affected by endotoxins [42,43]. The latter was confirmed by Nielsen et al., 2005, who reported that APR caused a negative nitrogen balance and a decreased functional liver mass that attenuated the urea synthesis increase [44]. In this regard, LPS severely affects the body’s nitrogen metabolism at the intestinal level [45]. Indeed, several studies have reported that endotoxemia is characterized by decreased amino acid intestinal absorption [42,43]. In addition, endotoxemia is linked to an increased release of amino acids from the muscles, such as glutamine, synthesized from glutamate and ammonia [43]. These amino acids are subsequently taken up by other cells, including hepatocytes and enterocytes of the small intestine, as a substrate for ATP production [43]. On the other hand, literature has reported that immune cells have higher glutamine requirements during inflammatory states such as sepsis, which could be responsible for changes in the body’s glutamine concentration [42]. Our results suggested that glutamate metabolism and urea cycle and their derivatives may have an essential role in calves’ diseases.

The other biochemical pathway that appeared to be affected by Gram-negative bacterial endotoxin was the folate metabolism (Figure 4). In this regard, Han et al., 2015 showed that the expression of the folate receptor was notably increased in lung macrophages, 48 h after intratracheal LPS. In other studies, low folate levels have been associated with various chronic inflammatory diseases, indicating that insufficient folate may occur because of inflammatory conditions or, consequently, that chronic inflammation increases folate requirements [46]. Moreover, it has been suggested that low folate status may play an important role in the neoplastic process [47]. As such, folate and its metabolic associations may serve as a valuable biomarker for calves’ disease diagnosis. In the current study, one major limitation relates to the metabolite identification, being a challenging process and still considered a major bottleneck in metabolomics research. Indeed, in this research, we were not able to achieve Tier 1 identification for our marker metabolites, although the candidate structures pointed towards certain chemical classes, which allowed perform biological interpretation and qualification of the biomarker panel. In this regard, it should be noted that using a single time-point for sampling after LPS challenge was somewhat a limitation, as well as multiple time-points would allow to in-depth assess the biological relevance of the biomarker signature as a whole and allow to describe the dynamics and relevance of the individual markers. A multi-matrix approach including blood, urine, and feces samples in future metabolomics studies related to calf’s studies could generate more insights in metabolite distribution, transformation, absorption, and clearance effects. In addition, in the current study, carboxylic acids and derivatives, and fatty acyls have the highest-class frequency, indicating that future research on lipidomics could be relevant to find new Gram-negative bacterial endotoxin’s biomarkers in calf’s diseases.

## 4. Materials and Methods

### 4.1. Calves, Treatments and Study Design

The experimental design was approved by the ethics committee of the Ferdowsi University of Mashhad, Iran (IRUMREC. #1399019). The in vivo experiment, treatments and APR evaluation were based on a prior study [24]. In brief, thirty bull calves, with a mean age of 34.8 ± 3.7 days and average body weight of 55.4 ± 4.3 kg, were housed outdoors in individual pens bedded with wheat straw at the facilities of the dairy farm of Astan Quds Razavi Animal Husbandry and Agriculture Co. (Mashhad, Iran) in February 2019. Calves were selected based on the type of delivery (without any difficulty), no record of disease or specific treatment, and quantitative criteria such as age and body weight, as well as a body temperature <39.5 °C. The duration of the actual experiment was 11 days (days 1–11), following a 7-day adaptation period (a week before day 1). On day 0 (a day before starting the experiment), calves were weighed (57.8 ± 4.5 kg) and randomly assigned to 1 out of 5 groups (six calves per group): (1) negative control (CON, the group which was subjected neither to LPS nor to other treatments), (2) positive control (LPS, injected once on day 8), (3) 350 mg/kg BW per day fish oil (days 1 to 11) + LPS (FO), (4) 4 mg/kg BW per day nanocurcumin (days 1 to 11) + LPS (CUR), and (5) 0.3 mg/kg BW dexamethasone (injected once, one hour before starting LPS challenge) + LPS (DEX). The sample size and practical setup were followed as proposed by Plessers et al., 2015 [5,19]. Calves were treated with 5 mg/kg BW enrofloxacin (RazakPharma Co., Tehran, Iran) a week before LPS challenge, to avoid unexpected respiratory infections. All groups received 5 L/d of whole milk and a diet based on NRC (2001) [48] recommendations, including a mixture of starter and chopped alfalfa hay. To prevent muscular dystrophy, twenty-five mg of vitamin E/PUFA (kg), was added to the milk. Fish oil (Kilka oil, Parskilka Sea Products Co., Mazandaran, Iran) and curcumin were mixed with the total milk and offered from day 1 until the end of the experiment (day 11). Curcumin oral capsules (SinaCurcumin, Exir Nano, Tehran, Iran), a registered product from curcuminoids (IRC: 1228225765), were used to increase the oral bioavailability of curcumin. On day 8, all experimental groups were intravenously challenged once with 0.5 μg/kg BW ultrapure LPS from *E. coli* serotype O111:B4 (Sigma–Aldrich^®^, Saint-Louis, Missouri; product NO. L2630), with exception of the CON group (intravenously injected with 0.5 μg/kg BW saline solution). To obtain the maximum efficiency of dexamethasone on the innate immune response, on day 8, one hour before the LPS challenge, the calves in group DEX were treated once with 0.3 mg/kg BW DEX (Aburaihan Pharmaceutical Co., Tehran, Iran) intramuscularly in the neck region (cervical ventral serratus muscle). Whenever calves would expose systemic shock symptoms, they would have been excluded from the experiment.

### 4.2. Acute Phase Response Evaluation and Sample Collection

To evaluate the effects of APR in calves, TNF-α, IL-6, RT, HR, and RR were recorded before and after LPS challenge. Blood samples were collected and transferred into EDTA-containing tubes to investigate the levels of TNF-α and IL-6. Following collection, the samples were centrifuged at 1000× *g* for 15 min at 4 °C to obtain plasma, after which samples were prediluted 10-fold and analyzed in duplicate, using commercially available ELISA kits (Bioassay Technology Laboratory ELISA kits, Shanghai, China; Cat No. E0019BO, and No. E0001BO for TNF-α and IL-6, respectively). The TNF-α and IL-6 assays resulted in intra-assay coefficients of variance (CV) for TNF-α and IL-6 of 9.6% and 9.2%, respectively. In the current study, it was opted to use fecal samples as non-invasive collection was possible, a higher number of metabolites compared to plasma [11] could be expected, and the fecal metabolome is known to uniquely integrate information on the host, exposomal factors (e.g., diet), and the gastrointestinal microbial community, with the latter being acknowledged to be involved in various (inflammatory) diseases. Fecal samples were acquired on day 0 (a day before starting the experiment) and day 9 (a day after LPS challenge). Fecal samples were collected by rectal stimulation, with sterile gloves to facilitate collection. For each calf, two fecal samples per day were collected, i.e., one before milk consumption in the morning and one in the afternoon. Both fecal samples were combined (equal amount on a wet weight basis) into one sample per calf per day. Finally, samples were lyophilized for 48 h and subsequently homogenized and sieved to attain representative aliquots, which were stored at −80 °C until analysis.

### 4.3. UHPLC-Q-Orbitrap-HRMS Based Metabolomic Evaluation

Extraction and UHPLC-Q-Orbitrap-HRMS measurements were performed according to De Paepe et al., 2018 [11]. In brief, ultrapure water and methanol were added as extraction solvents to 200 ± 0.5 mg of lyophilized feces. The supernatant was filtered using a polyamide filter (25 mm diameter, 0.45 μm pore size) (Machery-Nagel, Düren, Germany) and diluted with ultrapure water. Chromatographic separation was achieved by injecting a 10 µL extract aliquot in a Dionex UltiMate 3000 XRS UHPLC system (Thermo Fisher Scientific, San Jose, CA, USA), equipped with an Acquity HSS T3 column (Waters Corporation, 2.1 × 150 mm, 1.8 μm). The mobile phase consisted of ultrapure water (A) and acetonitrile (B), both acidified with formic acid (0.1% (*v*/*v*)), and was used to establish an 18-min gradient profile. Additionally, a fixed-mobile phase flow rate of 0.4 mL/min and a column oven temperature of 45 °C were set. MS analysis was performed using a Q-Exactive™ benchtop mass spectrometer (Thermo Fisher Scientific, San Jose, CA, USA), equipped with a heated electrospray ionization source (HESI-II) that was operated in polarity switching mode. Instrumental settings were a sheath gas flow rate of 50 arbitrary units (a.u.), auxiliary gas flow rate of 25 a.u., sweep gas flow rate of 5 a.u., a heater temperature of 350 °C, a capillary temperature of 250 °C, an S-lens RF level of 50%, and a spray voltage of ±2.7 kV. For full-scan analyses, the *m/z*-scan range was between 53.4 and 800 Da, mass resolution was 70,000 full width at half maximum (FWHM), automatic gain control (AGC) was 1 × 10^6^, and maximum injection time was 70 ms. Prior to analysis, ready-to-use calibration mixtures (Thermo Fisher Scientific, San Jose, CA, USA) were infused to calibrate the instrument, which warranted accurate mass measurements with deviations below 3 ppm. External standard mixtures (>100 polar to medium-polar target analytes) were injected to evaluate the operational conditions in terms of sensitivity and chromatographic performance [11]. Quality control (QC) samples were made from a pool composed of equal extract aliquots from all individual samples. Six QC samples were analyzed at the beginning of the analytical run to stabilize the system and in duplicate after each set of 10 biological samples to monitor instrumental fluctuations during analysis [49]. In total, 58 feces samples were analyzed.

### 4.4. Data Analysis

The statistical analyses of the APR experiment have been reported previously [24]. In brief, to evaluate APR in calves, data were first checked for normality, after which not-normally distributed data were analyzed using a Kruskal–Wallis test. Repeated-measures ANOVA was employed to evaluate the effect of LPS within the different groups. Data were analyzed in a randomized manner for the fixed effect of treatment, time and the interaction thereof, as well as the random effect of calf nested within treatment using JMP 13.2 software (SAS Institute Inc., Cary, NC, USA). Treatment significance (*p*-value < 0.05) was determined by the LSMEANS Tukey HSD test.

Establishment of the metabolic fingerprints was achieved using Compound Discoverer 3.0 (Thermo Fisher Scientific, San Jose, CA, USA) for positive and negative ionization mode simultaneously, with the following parameter settings: retention time tolerance 0.25 min, mass tolerance 6 ppm, *m*/*z* scan range 53.4–800 Da, retention time range 0.5–16 min, minimum peak intensity threshold 500,000 a.u., and S/N threshold 10. QC-samples and the total ion current (TIC) were used for ion abundance normalization adjust for both sample- and feature-specific analytical bias [23]. Metabolites that were significantly altered upon APR were selected by multivariate statistical analysis, using SIMCA^TM^ 15 software (Umetrics AB, Umea, Sweden), for which data were log-transformed to induce normality and pareto-scaled to reduce the relative importance of larger values. Multivariate statistical analysis was initiated by unsupervised principal component analysis (PCA-X) to visualize potential outliers and natural clustering. Subsequently, to reveal significant differences between LPS day 0 (LPS0) and LPS day 9 (LPS9) samples, orthogonal partial least squares discriminant analysis (OPLS-DA) models were constructed. Model-validity was verified based on the quality parameters R^2^X and Q^2^Y, cross-validated ANOVA (CV-ANOVA) (*p*-value < 0.05) and permutation testing (*n* = 100) [11]. APR-specific metabolites were selected based on the variable importance in projection (VIP > 1), Jack-knifed confidence interval (not across 0) and the S-plot descriptors (covariance and correlation). Finally, a *t*-test analysis within LPS group (day 0 vs. day 9) and CON group (day 0 vs. day 9) was performed to select the potential Gram-negative bacterial endotoxin markers.

### 4.5. Identification of Potential APR Metabolite Markers in Calves

Potential biomarkers (unknown metabolites) were tentatively identified based on the accurate *m*/*z*-value of the molecular ion, isotope profile (^13^C and ^34^S), and fragmentation MS-spectrum. With respect to the latter, parallel reaction monitoring MS/MS experiments were conducted, whereby the following settings were applied: *m*/*z* isolation width of 0.5 Da, mass resolution of 17,500 FWHM, AGC of 2 × 10^5^, and collision energies between 10 and 40 eV. Fragmentation data were analyzed using SIRIUS 4.0 software (Friedrich-Schiller University, Jena, Germany). For the chemical classification of potential biomarkers, we used ClassyFire a web-based application for automated structural classification of chemical entities [35].

The Mummichog algorithm [50] was used to evaluate altered biochemical pathways linked to Gram-negative bacterial endotoxins. Based on *m*/*z*, *p*-values and statistical scores of 3132 features ions obtained by the OPLS-DA model between LPS0 and LPS9, a likelihood list of affected pathways (*p*-value < 0.05) was obtained. The following settings were applied: a mass accuracy of 5 ppm, the mixed type for analytical mode, a *p*-value cutoff of 0.05, and the Bos Taurus (cow) [KEGG] library.

## 5. Conclusions

In conclusion, this study revealed various metabolite biomarkers that were significantly altered upon LPS-challenge and thus potentially involved in APR. Hereby, tentative annotation of these metabolite biomarkers revealed carboxylic acid derivatives—amino acids, peptides, and analogs—and fatty amides as the most prominent subclasses. As such, the annotated metabolite biomarkers, as well as other representatives from the cited subclasses, may enclose valuable potential for the diagnosis of various calf diseases. In addition, based on a more holistic interpretation of the metabolic fingerprint’s alterations, steroid, histidine, glutamate, and folate metabolism were also revealed as biochemical pathways that may play a role in Gram-negative bacterial endotoxins diseases in calves. However, future studies using multi-matrix metabolomics approaches including blood, urine, and feces samples are needed to generate more insights in metabolite distribution, transformation, absorption, and clearance effects of Gram-negative bacterial endotoxins in calf diseases.

## Figures and Tables

**Table 1:** The 37 Gram-negative bacterial endotoxin potential markers, retained after multivariate statistical analysis, with the assignment of a chemical formula using SIRIUS software.

In-House Identifier (ID)	*m*/*z*	RT (min)	Chemical Formula	Adduct Ion	VIP ^1^	*p*-Value ^2^	Average Fold-Change ^3^
1	88.11257	3.35	C_5_H_13_N	[M+H]^+^	3.02	0.00321	33.14
2	114.09153	1.11	C_6_H_11_NO	[M+H]^+^	2.07	0.02781	0.19
3	133.049116	1.94	C_3_H_8_N_3_O_3_	[M-H]^−^	1.95	0.04554	0.11
4	137.05927	7.65	C_6_H_8_N_3_O	[M-H]^−^	2.78	0.01925	0.02
5	146.11743	6.22	C_7_H_15_NO_2_	[M+H]^+^	2.42	0.02418	11.73
6	147.06314	9.31	C_4_H_8_N_3_O_3_	[M+H]^+^	1.97	0.02539	0.21
7	155.04495	4.13	C_6_H_6_N_2_O_3_	[M+H]^+^	2.09	0.03568	2.84
8	155.04480	1.86	C_4_H_6_N_5_O_2_	[M-H]^−^	2.11	0.02501	0.12
9	157.06067	1.85	C_6_H_8_N_2_O_3_	[M+H]^+^	2.18	0.00486	0.11
10	156.06519	4.74	C_5_H_9_N_4_O_2_	[M-H]^−^	2.14	0.00805	20.28
11	160.13307	7.47	C_8_H_17_NO_2_	[M+H]^+^	1.92	0.00807	5.90
12	165.05450	7.64	C_9_H_8_O_3_	[M+H]^+^	2.89	0.02354	0.02
13	165.02924	6.65	C_5_H_4_N_5_O_2_	[M-H]^−^	2.00	0.04593	0.17
14	179.09273	2.21	C_8_H_10_N_4_O	[M+H]^+^	2.34	0.02721	0.06
15	180.08516	2.21	C_4_H_11_N_4_O_4_	[M+H]^+^	2.02	0.04577	0.10
16	181.04941	7.65	C_7_H_8_N_3_O_3_	[M-H]^−^	2.69	0.02264	0.03
17	196.14429	6.77	C_10_H_17_N_3_O	[M+H]^+^	2.21	0.01398	15.15
18	205.13675	5.74	C_9_H_20_N_2_OS	[M+H]^+^	2.69	0.00040	20.51
19	215.17520	8.87	C_11_H_22_N_2_O_2_	[M+H]^+^	2.67	0.00220	18.92
20	221.16446	8.28	C_13_H_20_N_2_O	[M+H]^+^	2.83	0.00166	27.14
21 *	221.10532	7.05	C_12_H_15_NO_3_	[M-H]^−^	2.23	0.02531	3.44
22 *	227.05528	7.65	C_8_H_9_N_3_O_5_	[M-H]^−^	2.79	0.03370	0.03
23	229.19067	9.96	C_12_H_24_N_2_O_2_	[M+H]^+^	2.25	7.581 × 10^−6^	8.29
24	229.19089	10.34	C_12_H_24_N_2_O_2_	[M+H]^+^	2.31	0.00180	9.12
25	235.12117	7.97	C_11_H_16_N_4_O_2_	[M-H]^−^	2.80	0.00063	14.47
26	267.20618	6.22	C_15_H_26_N_2_O_2_	[M+H]^+^	2.09	0.02326	14.27
27 *	287.09198	10.33	C_15_H_9_N_7_	[M-H]^−^	1.89	0.00209	6.52
28	290.12280	6.09	C_12_H_19_NO_7_	[M+H]^+^	1.99	0.02156	3.09
29 *	313.14261	13.90	C_17_H_19_N_3_O_3_	[M+H]^+^	2.24	1.702 × 10^−6^	0.13
30	349.23645	11.38	C_21_H_32_O_4_	[M+H]^+^	1.93	0.01758	6.26
31 *	363.21713	11.45	C_21_H_32_O_5_	[M-H]^−^	2.30	0.00212	6.25
32 *	365.23264	11.37	C_21_H_34_O_5_	[M-H]^−^	2.32	0.00449	9.11
33	373.28003	7.04	C_18_H_36_N_4_O_4_	[M+H]^+^	1.90	0.01461	8.88
34	385.22260	11.11	C_20_H_34_O_7_	[M-H]^−^	2.15	0.00248	6.78
35 *	401.31131	7.963	C_20_H_40_N_4_O_4_	[M+H]^+^	2.34	0.00425	24.45
36 *	423.29312	7.96	C_17_H_38_N_6_O_6_	[M+H]^+^	2.08	0.01770	44.49
37	609.32690	11.13	C_33_H_44_N_4_O_7_	[M+H]^+^	1.88	0.02610	2.75

^1^ VIP = variable importance in projection, ^2^ *p*-value = *p*-value between LPS0 and LPS9 samples, ^3.^ Average fold-change = ratio between the average peak intensity in LPS9 samples (*n* = 5) and the average peak intensity in LPS0 samples (*n* = 5). * Represents IDs with their chemical formula determined based on MS^1^ only.

**Table 2:** Chemical identities, as proposed by SIRIUS software, for the top-ranked Gram-negative bacterial endotoxin candidate markers.

	Rank		
In-House Identifier (ID)	First	Second	Third
1	Amylamine	2-Aminopentane	Isoamylamine
2	*N*-Cyclobutylacetamide	*N*-(3-Butenyl)acetamide	*N*-Ethyloxolan-2-imine
3	*No candidate structures could be retrieved for the assigned chemical formula*		
4	Non-live (PubChem CID 57449799)	Non-live (PubChem CID 57424791)	Non-live (PubChem CID 57424804)
5	4-Acetamido-1-pentanol	*N*-[(S)-1-(Hydroxymethyl)butyl]acetamide	*N*-[(2R,4R)-4-Hydroxypentan-2-yl]acetamide
6	*No candidate structures could be retrieved for the assigned chemical formula*		
7	2-Carbamoyl-2-cyanocyclopropane-1-carboxylic acid	4-Nitrophenylhydroxylamine	4-Amino-2-nitrophenol
8	*No candidate structures could be retrieved for the assigned chemical formula*		
9	*N*-(Prop-2-enoylamino)oxyprop-2-enamide	4(3H)-Pyrimidinone, 6-hydroxy-2-methoxy-5-methyl-	[(*Z*)-C-Ethenyl-N-hydroxycarbonimidoyl] (1E)-N-hydroxyprop-2-enimidate
10	Oxoverdazyl	-	-
11	*N*-(1-Hydroxybutan-2-yl)-2-methylpropanamide	Ethyl [isopropyl(methyl)amino]acetat	2-(Hydroxymethyl)-N-propan-2-ylbutanamide
12	1-(3-Hydroxyphenyl)propane-1,2-dione	3-(3,5-Dihydroxyphenyl)prop-2-enal	(*E*)-3-(2,5-Dihydroxyphenyl)prop-2-enal
13	*No candidate structures could be retrieved for the assigned chemical formula*		
14	*N*,*N*-Dimethylimidazo[1,2-b]pyrazole-5-carboxamide	*N*,*N*-Dimethylimidazo[1,2-b]pyrazole-1-carboxamide	*N*-[(Dimethylamino)methylene]pyrazine-2-carboxamide
15	*No candidate structures could be retrieved for the assigned chemical formula*		
16	*No candidate structures could be retrieved for the assigned chemical formula*		
17	*N*-[2-(1H-Imidazol-5-yl)ethyl]-2,2-dimethylpropanamide	*N*-[2-(1H-Imidazol-5-yl)ethyl]pentanamide	Dolichotheline
18	(2*S*)-2-Amino-N-butan-2-yl-4-methylsulfanylbutanamide	(2*S*)-2-Amino-N-(4-methylsulfanylbutan-2-yl)butanamide	(2*S*)-2-Amino-N-(2-methylpropyl)-4-methylsulfanylbutanamide
19	*N*-(6-Acetamidohexyl)propanamide	*N*-(7-Acetamidoheptyl)acetamide	*N*-(4-Acetamidobutyl)pentanamide
20	2-Amino-N-(1-phenylpropan-2-yl)butanamide	*N*-Sec-Butyl-L-phenylalaninamide	(2*S*)-2-Amino-N-(1-phenylbutan-2-yl)propanamide
21	*No relevant fragmentation data could be acquired, for which structural elucidation was not possible*		
22	*No relevant fragmentation data could be acquired, for which structural elucidation was not possible*		
23	*N*-[(1R,4R)-4-(Propionylamino)-1-methylpentyl]propionamide	*N*-[5-(Propanoylamino)hexyl]propanamide	Leucyl-l-leucinal
24	6-Acetamido-N-(2-methylpropyl)hexanamide	6-Acetamido-N-butan-2-ylhexanamide	*N*-(5-Acetamidooctyl)acetamide
25	Non-live (PubChem CID 83431936)	Non-live (PubChem CID 83421718)	*N*-(2-Acetamidoethyl)-2-(methylamino)pyridine-3-carboxamide
26	5-Butyl-1,5-diisocyanatononane	1,9-Diisocyanato-5-methyl-5-propylnonane	2-[2-[Di(propan-2-yl)amino]ethoxy]-6-methoxyaniline
27	*No relevant fragmentation data could be acquired, for which structural elucidation was not possible*		
28	*N*-[(5R,6R,7S,8R)-6,7,8,9-Tetrahydroxy-2-methyl-3,4-dioxonon-1-en-5-yl]acetamide	[(2R,3S,4R,5R)-5-Acetamido-4-acetyloxy-2-hydroxy-6-oxohexan-3-yl] acetate	Triacetylmycosamine
29	*No relevant fragmentation data could be acquired, for which structural elucidation was not possible*		
30	7-[(1R,2S,5R)-2-Hydroxy-5-[(3S)-3-hydroxy-4-methyloct-1-en-6-ynyl]cyclopentyl]hept-5-enoic acid	(*Z*)-7-[(1R,2S,5R)-2-Hydroxy-5-[(E)-3-hydroxy-3-methyloct-1-en-6-ynyl]cyclopentyl]hept-5-enoic acid	6a-Carbaprostaglandin I3
31	*No relevant fragmentation data could be acquired, for which structural elucidation was not possible*		
32	*No relevant fragmentation data could be acquired, for which structural elucidation was not possible*		
33	6-[Bis[2-(2-methylpropylamino)-2-oxoethyl]amino]-N-hydroxyhexanamide	Leucylleucyllysine	2-[[2-(2,6-Diaminohexanoylamino)-4-methylpentanoyl]amino]-4-methylpentanoic acid
34	(*E*)-4,5-Dihydroxy-11-[3-(methoxymethyl)-4-oxooxetan-2-yl]-2,3,5,7-tetramethylundec-2-enoic acid	Methyl 11-(3-methoxymethyl-4-oxo-2-oxetanyl)-4,5-dihydroxy-3,5,7-trimethyl-2-undecenoate	5,6-Dihydroxyprostaglandin E1
35	*No relevant fragmentation data could be acquired, for which structural elucidation was not possible*		
36	*No relevant fragmentation data could be acquired, for which structural elucidation was not possible*		
37	*No candidate structures could be retrieved for the assigned chemical formula*		

## Data Availability

The data presented in this study are available on request from the corresponding author. The data are not publicly available due to privacy.

## Supplementary Materials

The following are available online at https://www.mdpi.com/2218-1989/11/2/108/s1, Table S1: Top-ranked chemical structures and their classification, as proposed by SIRIUS software for gram-negative bacterial endotoxin candidate markers, Table S2: The area under the curve for RT, HR, RR, TNF-α, and IL-6 in the LPS, DEX, FO, and CUR groups.
