# Supplementary material for: Metabolic Fingerprinting of Feces from Calves, Subjected to Gram-Negative Bacterial Endotoxin
Source: Metabolites. 2021 Feb 13;11(2):108. doi: 10.3390/metabo11020108 (PMC7918135; doi:10.3390/metabo11020108)
Supplement: Supplementary file 1 [file metabolites-11-00108-s001.pdf]

Supporting information

Metabolic Fingerprinting of Feces from Calves, Subjected to Gram Negative Bacterial Endotoxin

Saeid Kamel Oroumieh [1,2], Abbas Ali Naserian [2], Lieven Van Meulebroek[1], Ellen De Paepe[1], Reza Valizadeh[2], and Lynn Vanhaecke [1,*]

[1] Laboratory of Chemical Analysis, Department of Veterinary Public Health and Food Safety, Faculty of Veterinary Medicine, Ghent University, Salisburylaan 133, 9820 Merelbeke, Belgium;

[2] Department of Animal Science, Faculty of Agriculture, Ferdowsi University of Mashhad, P.O. Box 91775-1163, Mashhad, Iran;

* Correspondence: Lynn.Vanhaecke@Ugent.be; Tel.: +32- (0)9-264-7457

Table S1. Top-ranked chemical structures and their classification, as proposed by SIRIUS software for gram-negative bacterial endotoxin candidate markers.

ID	Rank	Name	PubChem CID	Superclass	Class	Subclass	Parent Level 1	Parent Level 2
1	1	Amylamine	8060	Organic nitrogen compounds	Organonitrogen compounds	Amines	Primary amines	Monoalkylamines
	2	2-Aminopentane	12246	Organic nitrogen compounds	Organonitrogen compounds	Amines	Primary amines	Monoalkylamines
	3	Isoamylamine	7894	Organic nitrogen compounds	Organonitrogen compounds	Amines	Primary amines	Monoalkylamines
2	1	N-Cyclobutylacetamide	23087721	Organic acids and derivatives	Carboxylic acids and derivatives	Carboxylic acid derivatives	Carboxylic acid amides	Acetamides
	2	N-(3-Butenyl)acetamide	13600051	Organic acids and derivatives	Carboxylic acids and derivatives	Carboxylic acid derivatives	Carboxylic acid amides	Acetamides
	3	N-Ethyloxolan-2-imine	21720742	Organoheterocyclic compounds	Oxolanes	-	Oxolanes	-
3		*No candidate structures could be retrieved for the assigned chemical formula*	-	-	-	-	-	-
4	1	Non-live	57449799	Organic nitrogen compounds	Organonitrogen compounds	Amines	Aralkylamines	-
	2	Non-live	57424791	Organoheterocyclic compounds	Diazines	Pyrazines	Methoxypyrazines	-

	3	Non-live	57424804	Organoheterocyclic compounds	Diazines	Pyrazines	Methoxypyrazines	-
5	1	4-Acetamido-1-pentanol	541465	Organic acids and derivatives	Carboxylic acids and derivatives	Carboxylic acid derivatives	Carboxylic acid amides	Acetamides
	2	N-[(S)-1-(Hydroxymethyl)butyl]acetamide	10464451	Organic acids and derivatives	Carboxylic acids and derivatives	Carboxylic acid derivatives	Carboxylic acid amides	Acetamides
	3	N-[(2R,4R)-4-Hydroxypentan-2-yl]acetamide	55299592	Organic acids and derivatives	Carboxylic acids and derivatives	Carboxylic acid derivatives	Carboxylic acid amides	Acetamides
6		*No candidate structures could be retrieved for the assigned chemical formula*	-	-	-	-	-	-
7	1	2-Carbamoyl-2-cyanocyclopropane-1-carboxylic acid	66771129	Organic acids and derivatives	Carboxylic acids and derivatives	Cyclopropanecarboxylic acids and derivatives	Cyclopropanecarboxylic acids	-
	2	4-Nitrophenylhydroxylamine	5486551	Benzenoids	Benzene and substituted derivatives	Nitrobenzenes	Nitrobenzenes	-
	3	4-Amino-2-nitrophenol	3417419	Benzenoids	Phenols	Nitrophenols	Nitrophenols	-
8		*No candidate structures could be retrieved for the assigned chemical formula*	-	-	-	-	-	-
9	1	N-(Prop-2-enoylamino)oxyprop-2-enamide	21319806	Organic acids and derivatives	Carboxylic acids and derivatives	Acrylic acids and derivatives	Acrylic acids and derivatives	-
	2	4(3H)-Pyrimidinone, 6-hydroxy-2-methoxy-5-methyl-	58035213	Organoheterocyclic compounds	Diazines	Pyrimidines and pyrimidine derivatives	Hydroxypyrimidines	-

	3	[(Z)-C-Ethenyl-N-hydroxycarbonimidoyl] (1E)-N-hydroxyprop-2-enimidate	88660874	Organic oxygen compounds	Organooxygen compounds	-	Organooxygen compounds	-
10	1	Oxoverdazyl	102285420	Organoheterocyclic compounds	Imidolactams	-	Imidolactams	-
	2	-	-	-	-	-	-	-
	3	-	-	-	-	-	-	-
11	1	N-(1-Hydroxybutan-2-yl)-2-methylpropanamide	43417884	Organic acids and derivatives	Carboxylic acids and derivatives	Carboxylic acid derivatives	Carboxylic acid amides	Secondary carboxylic acid amides
	2	Ethyl [isopropyl(methyl)amino]acetat	23465149	Organic acids and derivatives	Carboxylic acids and derivatives	Amino acids, peptides, and analogues	Amino acids and derivatives	Alpha amino acids and derivatives
	3	2-(Hydroxymethyl)-N-propan-2-ylbutanamide	117565592	Not found	Not found	Not found	Not found	Not found
12	1	1-(3-Hydroxyphenyl)propane-1,2-dione	17986404	Benzenoids	Benzene and substituted derivatives	Phenylpropanes	Phenylpropanes	-
	2	3-(3,5-Dihydroxyphenyl)prop-2-enal	54245519	Phenylpropanoids and polyketides	Cinnamaldehydes	-	Cinnamaldehydes	-
	3	(E)-3-(2,5-Dihydroxyphenyl)prop-2-enal	12023877	Phenylpropanoids and polyketides	Cinnamaldehydes	-	Cinnamaldehydes	-
13		*No candidate structures could be retrieved for the assigned chemical formula*	-	-	-	-	-	-
14	1	N,N-Dimethylimidazo[1,2-b]pyrazole-5-carboxamide	67821321	Not found	Not found	Not found	Not found	Not found

	2	N,N-Dimethylimidazo[1,2-b]pyrazole-1-carboxamide	19089098	Organoheterocyclic compounds	Azoles	Imidazoles	Substituted imidazoles	Carbonylimidazoles
	3	N-[(Dimethylamino)methylene]pyrazine-2-carboxamide	16727655	Organoheterocyclic compounds	Diazines	Pyrazines	Pyrazine carboxylic acids and derivatives	Pyrazinecarboxamides
15		*No candidate structures could be retrieved for the assigned chemical formula*	-	-	-	-	-	-
16		*No candidate structures could be retrieved for the assigned chemical formula*	-	-	-	-	-	-
17	1	N-[2-(1H-Imidazol-5-yl)ethyl]-2,2-dimethylpropanamide	21360995	Organoheterocyclic compounds	Azoles	Imidazoles	Imidazoles	-
	2	N-[2-(1H-Imidazol-5-yl)ethyl]pentanamide	24284602	Lipids and lipid-like molecules	Fatty Acyls	Fatty amides	N-acyl amines	-
	3	Dolichotheline	168082	Lipids and lipid-like molecules	Fatty Acyls	Fatty amides	N-acyl amines	-
18	1	(2S)-2-Amino-N-butan-2-yl-4-methylsulfanylbutanamide	61148528	Not found	Not found	Not found	Not found	Not found
	2	(2S)-2-Amino-N-(4-methylsulfanylbutan-2-yl)butanamide	103797251	Organic acids and derivatives	Carboxylic acids and derivatives	Amino acids, peptides, and analogues	Amino acids and derivatives	Alpha amino acids and derivatives
	3	(2S)-2-Amino-N-(2-methylpropyl)-4-methylsulfanylbutanamide	22691833	Organic acids and derivatives	Carboxylic acids and derivatives	Amino acids, peptides, and analogues	Amino acids and derivatives	Alpha amino acids and derivatives
19	1	N-(6-Acetamidohexyl)propanamide	58534772	Organic acids and derivatives	Carboxylic acids and derivatives	Carboxylic acid derivatives	Carboxylic acid amides	Acetamides
	2	N-(7-Acetamidoheptyl)acetamide	340007	Organic acids and derivatives	Carboxylic acids and derivatives	Carboxylic acid derivatives	Carboxylic acid amides	Acetamides

	3	N-(4-Acetamidobutyl)pentanamide	110602639	Lipids and lipid-like molecules	Fatty Acyls	Fatty amides	N-acyl amines	-
20	1	2-Amino-N-(1-phenylpropan-2-yl)butanamide	63011958	Carboxylic acids and derivatives	Amino acids, peptides, and analogues	Amino acids and derivatives	Alpha amino acids and derivatives	Organic acids and derivatives
	2	N-Sec-Butyl-L-phenylalaninamide	12968595	Carboxylic acids and derivatives	Amino acids, peptides, and analogues	Amino acids and derivatives	Alpha amino acids and derivatives	Organic acids and derivatives
	3	(2S)-2-Amino-N-(1-phenylbutan-2-yl)propanamide	119313535	Benzene and substituted derivatives	Phenethylamines	Amphetamines and derivatives	Benzenoids	-
21		*No relevant fragmentation data could be acquired, for which structural elucidation was not possible*	-	-	-	-	-	-
22		*No relevant fragmentation data could be acquired, for which structural elucidation was not possible*	-	-	-	-	-	-
23	1	N-[(1R,4R)-4-(Propionylamino)-1-methylpentyl]propionamide	12102040	Organic acids and derivatives	Carboxylic acids and derivatives	Carboxylic acid derivatives	Carboxylic acid amides	Secondary carboxylic acid amides
	2	N-[5-(Propanoylamino)hexyl]propanamide	58779522	Organic acids and derivatives	Carboxylic acids and derivatives	Carboxylic acid derivatives	Carboxylic acid amides	Secondary carboxylic acid amides
	3	Leucyl-l-leucinal	484685	Organic acids and derivatives	Carboxylic acids and derivatives	Amino acids, peptides, and analogues	Amino acids and derivatives	Alpha amino acids and derivatives
24	1	6-Acetamido-N-(2-methylpropyl)hexanamide	60653476	Lipids and lipid-like molecules	Fatty Acyls	Fatty amides	N-acyl amines	-

	2	6-Acetamido-N-butan-2-ylhexanamide	60652726	Lipids and lipid-like molecules	Fatty Acyls	Fatty amides	N-acyl amines	-
	3	N-(5-Acetamidooctyl)acetamide	57543871	Organic acids and derivatives	Carboxylic acids and derivatives	Carboxylic acid derivatives	Carboxylic acid amides	Acetamides
25	1	Non-live	83431936	Organoheterocyclic compounds	Imidazopyridines	-	Imidazopyridines	-
	2	Non-live	83421718	Organoheterocyclic compounds	Imidazopyridines	-	Imidazopyridines	-
	3	N-(2-Acetamidoethyl)-2-(methylamino)pyridine-3-carboxamide	55139084	Organoheterocyclic compounds	Pyridines and derivatives	Pyridinecarboxylic acids and derivatives	Pyridinecarboxamides	Nicotinamides
26	1	5-Butyl-1,5-diisocyanatononane	87324424	Organic nitrogen compounds	Organonitrogen compounds	Isocyanates	Isocyanates	-
	2	1,9-Diisocyanato-5-methyl-5-propylnonane	54160877	Not found	Not found	Not found	Not found	Not found
	3	2-[2-[Di(propan-2-yl)amino]ethoxy]-6-methoxyaniline	104260349	Benzenoids	Phenol ethers	Aminophenyl ethers	Aminophenyl ethers	-
27		*No relevant fragmentation data could be acquired, for which structural elucidation was not possible*	-	-	-	-	-	-
28	1	N-[(5R,6R,7S,8R)-6,7,8,9-Tetrahydroxy-2-methyl-3,4-dioxonon-1-en-5-yl]acetamide	23588580	Organic oxygen compounds	Organooxygen compounds	Carbohydrates and carbohydrate conjugates	Aminosaccharides	-
	2	[(2R,3S,4R,5R)-5-Acetamido-4-acetyloxy-2-hydroxy-6-oxohexan-3-yl] acetate	18610468	No data available	No data available	No data available	No data available	No data available
	3	Triacetylmycosamine	90479594	Not found	Not found	Not found	Not found	Not found

29		*No relevant fragmentation data could be acquired, for which structural elucidation was not possible*	-	-	-	-	-	-
30	1	7-[(1R,2S,5R)-2-Hydroxy-5-[(3S)-3-hydroxy-4-methyloct-1-en-6-ynyl]cyclopentyl]hept-5-enoic acid	57090564	No data available	No data available	No data available	No data available	No data available
	2	(Z)-7-[(1R,2S,5R)-2-Hydroxy-5-[(E)-3-hydroxy-3-methyloct-1-en-6-ynyl]cyclopentyl]hept-5-enoic acid	70588329	No data available	No data available	No data available	No data available	No data available
	3	6a-Carbaprostaglandin I3	6438653	No data available	No data available	No data available	No data available	No data available
31		*No relevant fragmentation data could be acquired, for which structural elucidation was not possible*	-	-	-	-	-	-
32		*No relevant fragmentation data could be acquired, for which structural elucidation was not possible*	-	-	-	-	-	-
33	1	6-[Bis[2-(2-methylpropylamino)-2-oxoethyl]amino]-N-hydroxyhexanamide	11222529	Organic acids and derivatives	Carboxylic acids and derivatives	Amino acids, peptides, and analogues	Amino acids and derivatives	Alpha amino acids and derivatives
	2	Leucylleucyllysine	18222159	Organic acids and derivatives	Carboxylic acids and derivatives	Amino acids, peptides, and analogues	Peptides	Oligopeptides
	3	2-[[2-(2,6-Diaminohexanoylamino)-4-methylpentanoyl]amino]-4-methylpentanoic acid	18222510	Organic acids and derivatives	Carboxylic acids and derivatives	Amino acids, peptides, and analogues	Peptides	Oligopeptides
34	1	(E)-4,5-Dihydroxy-11-[3-(methoxymethyl)-4-oxooxetan-2-yl]-2,3,5,7-tetramethylundec-2-enoic acid	88601011	Lipids and lipid-like molecules	Fatty Acyls	Fatty acids and conjugates	Long-chain fatty acids	-
	2	Methyl 11-(3-methoxymethyl-4-oxo-2-oxetanyl)-4,5-dihydroxy-3,5,7-trimethyl-2-undecenoate	54523016	Not found	Not found	Not found	Not found	Not found
	3	5,6-Dihydroxyprostaglandin E1	6444125	No data available	No data available	No data available	No data available	No data available
35		*No relevant fragmentation data could be acquired, for which structural elucidation was not possible*	-	-	-	-	-	-

36	*No relevant fragmentation data could be acquired, for which structural elucidation was not possible*	-	-	-	-	-	-
37	*No candidate structures could be retrieved for the assigned chemical formula*	-	-	-	-	-	-

Table S2. The area under the curve for RT, HR, RR, TNF-α, and IL-6 in the LPS, DEX, FO, and CUR groups.

	Groups[1]					
Item	LPS	FO	CUR	DEX	SEM	*P*-Value
RT[2]	937.4	936.1	937.7	935.3	1.44	0.68
HR[3]	2364	2424	2493	2300	151.6	0.58
RR[4]	336.8	332.8	329.2	318.6	27.1	0.89
TNF-α[5]	23.9^{a}	21.6^{a}	20.8^{a}	9.2^{b}	3.43	<0.01
IL-6[6]	57.7^{a}	54.3^{a}	52.8^{a}	16.9^{b}	7.35	<0.01

^{a,b} Values with different superscripts are significantly different ($P < 0.01$).

[1] Groups: LPS = positive control; FO = 350 mg/kg BW per day fish oil + LPS; CUR = 4 mg/kg BW per day nanocurcumin + LPS; DEX = 0.3 mg/kg BW dexamethasone + LPS. [2] RT = rectal temperature, AUC$_{0-24h}$ (°C × h). [3] HR = heart rate, AUC$_{0-24h}$ (bpm × h). [4] RR = respiratory rate, AUC$_{0-6h}$ (bpm × h). [5] TNF-α = tumor necrotic factor α, AUC$_{0-4h}$ (ng/mL × h). [6] IL-6 = interleukin-6, AUC$_{0-6h}$ (ng/mL × h).
